# Progressive Occlusion of Enterprise Stent-Assisted Coiling of Ruptured Wide-Necked Intracranial Aneurysms and Related Factors on Angiographic Follow-Up: A Single-Center Experience with 468 Patients

**DOI:** 10.1371/journal.pone.0092407

**Published:** 2014-03-21

**Authors:** Tangming Peng, Zenghui Qian, Aihua Liu, Youxiang Li, Chuhan Jiang, Zhongxue Wu

**Affiliations:** Department of Interventional Neuroradiology, Beijing Neurosurgical Institute, Beijing Tiantan Hospital, Capital Medical University, Beijing, China; University Medical Center (UMC) Utrecht, Netherlands

## Abstract

This study was designed to assess the effect of the Enterprise stent on progressive occlusion of wide-necked aneurysms and to evaluate the association between dubious factors and progressive occlusion, which is a consecutive, retrospective, single-center study. Data from 468 patients with 495 wide-necked aneurysms, who had undergone Enterprise stent-assisted coiling (SAC) were reviewed, and the clinical outcomes and the angiographic results were analyzed. A 14-month clinical follow-up was achieved in 421 of the 468 patients (90.0%), showing modified Rankin Scale (mRS) 0–1 in 364 (86.4%), mRS 2 in 17 (4.1%), mRS 3 in 17 (4.1%), mRS 4–5 in 9 (2.1%), and mRS 6 in 14 (3.3%) patients. Overall, the morbidity and mortality were 10.2% and 3.3%, respectively. Initial angiographic results showed Raymond scale (RS)1 in 273 (55.2%), RS2 in 194 (39.2%), and RS3 in 28 (5.6%) patients. Eight-month angiographic follow-up was available in 394 of 495 patients (79.6%), and RS1 was seen in 315 (79.9%), RS2 in 65 (16.5%) and RS3 in 14 (3.6%) cases. At the end of the follow-up, 115 of the 165 (69.7%) patients with initial RS2 and RS3 showed progressive occlusion. Statistical analysis showed no significant difference between progressive occlusion and age (p = 0.654), sex (p = 0.016), aneurysm diameter (p = 0.010), neck size (p = 0.124), dome-to neck ratio (DNR) (p = 0.018) and location (p = 0.001) at the time of follow-up. SAC using Enterprise stent is not only feasible for wide-necked aneurysms, but can achieve a high rate of progressive occlusion with good clinical outcomes at medium-term follow-up. Patient age and aneurysm neck size showed no associated with progressive occlusion at follow-up, while sex, aneurysm diameter, DNR and location were significantly associated with progressive occlusion.

## Introduction

Since the publication of the results of the International Subarachnoid Aneurysm Trial, endovascular embolization of intracranial aneurysms has been firmly established as an effective treatment in patients with aneurysms. However, endovascular embolization of wide-necked intracranial aneurysms remains a technical challenge, especially when the anatomy is complex. Several devices have been designed to assist coiling of wide-necked intracranial aneurysms. Despite significant advancements in coil and balloon remolding [1], [2], the risk of coil protrusion into the parent vessel is inevitable, while SAC can prevent coil herniation[3]. In addition, intracranial stents might also prevent aneurysm regrowth and reduce the aneurysm recanalization rate. Enterprise Vascular and Delivery System (Codman Neurovascular, Miami Lakes, FL, USA), a self-expanding, closed-cell coil that has been especially designed for wide-necked intracranial aneurysms, represents a significant improvement in SAC of wide-necked aneurysms previously considered not amenable to embolization or clipping and has been available since 2009 in our institution. After approval by the US Food and Drug Administration, many studies have investigated the safety and efficacy of Enterprise SAC. However, there is no published report on single or multiple aneurysms evaluating the progressive occlusion rate of wide-necked intracranial aneurysms using Enterprise stent.

This study was designed to evaluate the effect of the Enterprise stent on progressive occlusion of wide-necked intracranial aneurysms and to evaluate the impact of factors on progressive occlusion of angiographic follow-up results in these patients.

## Materials and Methods

### Ethics Statement

The study protocol was approved by the Institutional Review Board at the Beijing Tiantan Hospital. All patients gave written informed consent to participate and the privacy of patients was strictly protected.

### Study Design

A retrospective database was established based on the review of records from all patients with ruptured wide-necked intracranial aneurysms who had undergone endovascular embolization with SAC between April 2009 and December 2012. A total of 468 patients with Enterprise SAC for wide-necked intracranial aneurysms were included. The decision whether to use Enterprise SAC or to clip the aneurysm depended on consensus between a vascular neurosurgeon and a neurointerventional specialist. A wide neck was defined as aneurysm neck greater than 4 mm in diameter or an aneurysm with a neck diameter smaller than 4 mm with a dome to neck ratio of less than 2. The Hunt and Hess grade was applied for patients with ruptured aneurysms to evaluate the neurological condition at the time of admission.

### Pharmacology Management

All patients received an initial heparin bolus administration (70–100 UI/kg) followed by a continuous infusion, which was used for continuous rinsing of the catheters, and the activated clotting time was maintained between 250 and 300 seconds during the procedure. A loading dose of clopidogrel and aspirin (300 mg each) was administered orally or rectally 2 hours before the stent was to be deployed for patients with ruptured aneurysms, while patients with unruptured aneurysms received a loading dose of clopidogrel and aspirin (100 mg each) orally three days before the procedure. After the procedure, all patients were maintained on daily doses of clopidogrel and aspirin (100 mg each) for 4 weeks, followed by aspirin alone (100 mg daily) for another five months. In 2012, a patient encountered a delayed in-stent stenosis using the Enterprise stent. The patient had cytochrome P450 2C19 (CYP2C19) “2 and 3” alleles, resulting in a contradiction to clopidogrel treatment. Therefore, CYP2C19 was required to examine all the patients who were taking antiplatelets.

### Endovascular Therapy Procedures

A conventional bilateral carotid artery and vertebral artery and three-dimensional rotational angiography were performed to evaluate the aneurysm features and parent vessel condition before stent introduction. All patients were treated with the Enterprise Vascular and Delivery System only. Stents were chosen allowing for 5-mm coverage on either side of the aneurysm neck. A variety of coiling techniques were used for the stent coiling, including the sequential, jailing and waffle cone techniques. In the sequential technique, the Enterprise stent was first deployed to cover the neck of the aneurysm. Then, a coil microcatheter was gently introduced into the aneurysm sac through the mesh of the stent, through which the coils were delivered one by one until the coil mesh was stable in the sac. In the later technique, a separate microcatheter was placed in the aneurysm sac, and the first coil was then introduced into the sac (not detached) followed by the stent that was deployed parallel to the microcatheter between the stent and vessel wall for stabilization. At last, the subsequent coils were introduced into the sac until ideal packing was achieved. In the third technique, the stent was designed to construct an artificial neck in a basilar artery terminal aneurysm, and the markers were placed on the proximal portion of the aneurysm, which was followed by coiling. After SAC was achieved, a conventional angiography was performed to access the initial occlusion rate.

### Clinical Evaluation

Clinical evaluations were performed before and after treatment, at discharge, and at 6–49 month (average 14 month) follow-up. Clinical outcomes of SAC were evaluated using the mRS and the neurologic conditions, which were determined at follow-up and compared with the patients’ initial neurologic status. A good clinical outcome was defined as a mRS score of 0–2 at the time of follow-up.

### Anatomic Evaluation

Angiographic result was predefined based on radiographic criteria. Initial and follow-up angiographic images were performed to evaluate the progressive occlusion rate of the aneurysms. The average duration of angiographic follow-up was 8.2 month, ranging from 3 to 34 months. The angiographic results were evaluated with the Raymond grading system as follows: complete occlusion (Raymond Scale 1), residual neck (Raymond Scale 2) and residual aneurysm (Raymond Scale 3).

### Data Collection

Data on patient demographics, neurologic conditions, and aneurysm features were collected based on the records of all patients with wide-necked aneurysms who had received an Enterprise stent. Angiographic outcomes included the initial and follow-up aneurysm occlusion rates. Clinical outcomes were collected using the documented mRS score.

### Statistical Analysis

SPSS 19.0 was used to calculate the statistical results. Means and standard deviations (SD) were provided for continuous variables, and Student’s t-test was used for these variables if they met the normal distribution. Rank sum test, Fisher’s exact test or the chi-squared test were used for analysis of the categorical variables when appropriate. A P value <0.05 was regarded as statistical significant.

## Results

### Patient Population

From April 2009 to December 2012, 468 patients underwent SAC of wide-necked aneurysms using Enterprise stent. Of the 468 patients, 308 were female (65.8%) and 165 were male (34.2%), and the average age was 53.4±11.1 years, ranging from 15 to 79 years. A total of 122 patients (26.1%) had ruptured wide-necked aneurysms, and 346 patients (73.9%) had unruptured wide-necked aneurysms. Single aneurysms were observed in 386 patients (82.4%). In addition, 68 patients (14.5%) had 134 aneurysms, 12 patients (2.6%) had 36 aneurysms, and 2 patients (0.4%) had 8 aneurysms. Among the 566 aneurysms, 510 aneurysms were treated with endovascular treatment, and Enterprise stent-assisted coiling was performed on 495 aneurysms.

### Aneurysm Characteristics

Among the 495 aneurysms that had been treated with Enterprise SAC, mean aneurysm size ± standard deviation was 6.51±4.89 mm (range, 1.5–38 mm), 51.1% of the aneurysms were <5 mm (n = 253), 31.9% ranged between 5 and 9.9 mm (n = 158), and 15.8% ranged between 10 and 24.9 mm (n = 78). In addition, a dome size larger than 25 mm was seen in six aneurysms (1.2%).

The mean diameter of the aneurysm neck size ± standard deviation was 6.93±4.77 mm (range, 1.5–34 mm). In all the aneurysms, the dome-to-neck ratio ± standard deviation was 0.99±0.50 (range, 0.13–7.19). In the aneurysms with a neck size smaller than 4 mm, DNR was 1.07±0.27 (range, 0.62–1.95), and DNR of the aneurysms with a neck size larger than 4 mm was 0.96±0.55 (range, 0.13–7.19). The most common locations of the 495 aneurysms were the internal carotid artery (251/495, 50.7%), followed by the posterior communicating artery (107/495, 21.6%), the vertebral artery (83/495, 16.8%), the basilar artery (30/495, 6.1%), the middle cerebral artery (11/495, 2.2%), the posterior cerebral artery (7/495, 1.4%), the anterior cerebral artery and the anterior communicating artery (6/495, 1.2%).

### Anatomic Results of Initial and Follow-up Angiography

Initial angiographic results after the procedure showed that RS1 was achieved in 273 aneurysms (55.2%), RS2 in 194 aneurysms (39.2%), and RS3 in 28 aneurysms (5.6%). Angiographic follow-up was available in 371 of 468 patients (79.3%) with an average of 8.2 months, with a range from 3 to 34 months, and a total of 394 of 495 aneurysms (79.6%) were seen in follow-up. Among the 394 aneurysms, 163 aneurysms were followed-up for 3 to 6 months, 201 aneurysms for 7 to 12 months, and the remained 30 aneurysms were followed-up for over 12 months. The angiographic follow-up results of 394 aneurysms showed RS1 in 315 (79.9%), RS2 in 65 (16.5%), and RS3 in 14 aneurysms (3.6%) (Table 1), and the angiographic follow-up results of the three periods are shown in table 2. The evolution of 394 aneurysms showed that 208 of 229 aneurysms (90.8%) initially in RS1 remained unchanged, while 19 (8.3%) deteriorated to RS2 with a neck remnant, and 2 aneurysms deteriorated to RS3 (0.9%). Ninety-nine of 146 (69.7%) RS2 progressed to RS1, 38 (26.0%) remained unchanged, and 9 (6.2%) deteriorated to RS3. In the remaining 19 aneurysms that were initially RS3, 8 (42.1%) progressed to RS1 while 3 aneurysms (15.8%) remained unchanged (Table 3). Consequently, in the 165 aneurysms that initially had incomplete occlusion (RS2 and RS3), 115 aneurysms (69.7%) achieved a progressive occlusion rate, while 9 aneurysms (5.5%) deteriorated.

**Table 1:** Initial and follow-up angiographic results of 495 aneurysms with Enterprise SAC.

Raymond Class	Initial		follow-up		P value
	N	%	N	%	0.034
Raymond Scale 1	273	55.2	315	79.9	
Raymond Scale 2	194	39.2	65	16.5	
Raymond Scale 3	28	5.6	14	3.6	
Total	495		394		

**Table 2:** Angiographic follow-up results at 3–6 months, 7–12 months and over 12 months.

Raymond Class	Initial		follow-up at 3–6M		follow-up at 7–12M		follow-up >12M		follow-up for all patients	
	N	%	N	%	N	%	N	%	N	%
Raymond Scale 1	273	55.2	137	84.0	156	77.6	22	73.3	315	79.9
Raymond Scale 2	194	39.2	22	13.5	37	18.4	6	20	65	16.5
Raymond Scale 3	28	5.6	4	2.5	8	4.0	2	6.7	14	3.6
Total	495		163		201		30		394	

**Table 3:** Follow-up angiographic results of 394 aneurysms with Enterprise SAC.

Raymond Scale		N	%
Progress	3–2	8	2.0
	3–1	8	2.0
	2–1	99	25.1
unchanged	1	208	52.8
	2	38	9.6
	3	3	0.7
worsen	1–2	19	4.8
	2–3	9	2.3
	1–3	2	0.5
Total		394	100

### Clinical outcomes

Clinical follow-up was achieved in 421 of 468 patients (90.0%) with an average of 14 months and a range from 6 to 49 months. At the end of follow-up, mRS score of 0–1 was achieved in 364 cases (86.4%), mRS score of 2 in 17 (4.1%), mRS score of 3 in 17 (4.1%), mRS score of 4–5 in 9(2.1%), and mRS score of 6 in 14 cases (3.3%). Therefore, the morbidity and mortality were 10.2% and 3.3%, respectively.

### Recurrence and Subsequent Embolization of Aneurysms

Among the 394 aneurysms with angiographic follow-up, 30 aneurysms had a deteriorated occlusion rate (Table 3). Therefore, the incidence of wide-necked aneurysms treated with SAC using Enterprise stent was 7.6%. In our patients with recurrence, 14 aneurysms in RS3 were re-treated with additional coiling without stenting, while the aneurysms with a neck residual were strictly followed-up.

### Statistics Analysis Results

Angiographic follow-up results were divided into two groups according to the occlusion rate, and included the complete occlusion group and the incomplete occlusion group. The relationship between angiographic follow-up results and patient demographic and aneurysm features are shown in Table 4.

**Table 4:** Effects of clinical factors on angiographic result at follow-up.

	Complete occlusion group	Incomplete occlusion group	P value
Age	53.0±11.1	53.6±10.3	0.654
Sex M	94	32	0.016
F	208	37	
Aneurysm diameter	6.1±4.5	7.7±6.1	0.010
Neck size	6.6±4.5	7.5±5.3	0.124
Dome-to-neck ratio	0.97±0.46	1.12±0.64	0.018
Location			
PC	59	28	0.001
AC	256	51	

## Discussion

The present study was designed to evaluate the progressive occlusion rate of Enterprise SAC in wide-necked aneurysms in a single center experience. The confounding factors were excluded, including aneurysms with an arteriovenous malformation, dual arteriovenous fistulae, and moyamoya disease. When comparing previous studies on SAC in wide-necked aneurysms (ruptured and unruptured), our study resulted in a higher progressive occlusion rate at midterm follow-up. Nevertheless, slight discrepancies were observed and discussed below.

Our study confirmed that Enterprise stent placement is not only feasible for treatment of wide-necked aneurysms, but it also confirmed the high rate of progressive occlusion (69.7%) in RS2 and RS3 at short-midterm follow-up. In addition, the clinical factors, including age and neck size were not statistically associated with the aneurysm occlusion rate at the end of follow-up, while sex, the aneurysm diameter, the dome-to-neck ratio and the aneurysm location showed significant difference between the complete occlusion group and incomplete occlusion group at follow up.

Endovascular treatment of wide-necked, large or giant aneurysms with protection of the parent artery is technically challenging [4]–[6]. Detachable coils and vascular remodeling, which offers these patients a treatment, has been under development. Two different techniques have been approved for vascular remodeling. The first technique involves temporary parent artery occlusion with a non-detachable balloon, which is used to remodel the aneurysm neck [7]. The second technique involved deployment of an intracranial stent, such as Enterprise stent, across the aneurysm neck and subsequent coil introduction into the aneurysm sac through the stent mesh, which prevents their misplacement into the vessel lumen [8]. SAC reduces the aneurysm recurrence rate and the need for subsequent re-treatment. It is interesting to note that the mean size of the aneurysms in our series (6.51 mm) was lower than in previous studies [9]–[13] (mean range, 6.7–13.2 mm), and 51.1% of the aneurysms (253 of 495) in our series were smaller than 5 mm. In addition, 411 aneurysms (83.3%) were smaller than 10 mm. This phenomenon most likely reflected that SAC was more widely performed using Enterprise stent, which was initially reserved for wide-necked aneurysms.

Previous reports of embolization of aneurysms using the Neuroform stent (Boston Scientific, USA) showed that initial angiographic occlusion was poor due to the inherent difficulty of crossing the stent struts on the first attempt or in repeated attempts of gaining access to the aneurysm. Enterprise Vascular Reconstruction Device and Delivery System had a high rate of ideal navigation and successful deployment. The published initial angiographic results of endovascular embolization of wide-necked aneurysms (more than 30 aneurysms) using Enterprise stent were extremely variable, with initial complete occlusion rates ranging from 19% to 56% [10], [11], [14]–[17]. In the present study, the initial complete occlusion rate was 55.2%, which was clear to the highest reported rate.

Weber et al. [15] were the first to report the progressive occlusion rate of 31 unruptured wide-necked aneurysms using Enterprise SAC. The progressive occlusion rate of aneurysms in our series is higher than the rate reported by Weber et al, who presented 6 months of angiographic follow-up data of 31 saccular, wide-necked intracranial aneurysms using Enterprise SAC. They reported that the progressive occlusion was achieved in 10 of 25 patients (40%) with initial incomplete occlusion aneurysms, and the complete occlusion was achieved in 16 of 31 patients (51.6%) at the time of follow-up. Therefore, the initial complete occlusion rate (19.4%, 6 of 31), the progressive occlusion rate (40%) and the follow-up complete occlusion rate (51.6%) were lower than in our series (55.2%, 69.7% and 79.9%) with a mean 8.2-month follow-up. Recently, Fargen et al [17] reported the largest series of patients with single aneurysms (229 patients with 229 aneurysms). The 21-month angiographic follow-up results showed that the complete occlusion rate (59%) was also better than that reported by Weber et al, while they did not show the initial complete occlusion rate and the progressive occlusion rate. However, the report by Fargen showed that the mean aneurysm size (9.2±0.4 mm) was larger than the one reported by Weber et al (7±4 mm). Therefore, the duration of long-term follow-up may be a factor contributing to the differences between studies regarding the reported rate of complete occlusion and progressive occlusion.

Compared with previous Neuroform studies, progressive occlusion rates were extremely variable (ranging from 14–57.1%) [18]–[21], whereas progressive occlusion was observed in 69.7% of patients in our series with ruptured aneurysms, which had been regarded as a higher progressive occlusion rate in previous studies. The high rate of progressive occlusion may be explained by the technical and physiological properties of the Enterprise stent itself. The Enterprise stent may also promote progressive thrombosis and offer scaffolding for reconstruction of the intimal layer of the parent artery at the aneurysm neck with respect to the mechanical, hemodynamic, and biological benefits provided by Enterprise. These benefits can prevent coil protrusion into the parent artery, produce flow redirection, reduce intra-aneurysmal blood flow, and promote vessel wall healing [22], [23].

In comparison, the previous published studies with 14-month clinical outcomes, the good clinical outcome (86.4%, mRS score of 0–1), the morbidity (10.2%, mRS score of 2–4) and mortality (3.3%, mRS score of 6) in our series showed that SAC of wide-necked aneurysms using Enterprise stents was safe and efficacious. In addition, effects of patient demographic and aneurysm features on short- and mid-term durability of SAC of wide-necked aneurysms using Enterprise stent had not been calculated and analyzed before. In the present study, age and aneurysm neck size were not significantly different to progressive occlusion at follow-up, while sex, aneurysm dome size, dome-to-neck ratio and aneurysm location (AC versus PC) showed a significant difference between the groups, which may also indicate that the aneurysm features and the Enterprise stent promote the progressive occlusion.

There are several limitations to the present study, including the retrospective nature, the sample of patients, and the limited follow-up duration, and the lack of logistic analysis of factors. Nevertheless, due to the fact that this study was designed to evaluate the endovascular embolization of single wide-necked aneurysms using Enterprise stents, the results of this study may serve as a reference for such aneurysms and provide the basis for future studies.

## Conclusion

SAC using Enterprise stents is not only feasible for wide-necked aneurysms, but can achieve a high rate of progressive occlusion with good clinical outcomes at medium-term follow-up. Patient age and neck size showed no association with progressive occlusion at follow-up, while sex, aneurysm dome size, dome-to-neck ratio and location were significantly associated with progressive occlusion.
